# Responsive Neurostimulation in Patients with a History of Viral Brain Infections—A Single-Center Experience

**DOI:** 10.3390/neurosci7030068

**Published:** 2026-06-13

**Authors:** Melissa Huynh Mabry, Irina Podkorytova, Ebenezer Chinedu-Eneh, Sasha Alick-Lindstrom, Kan Ding, Ryan Hays, Ghazala Perven

**Affiliations:** Department of Neurology, University of Texas Southwestern Medical Center, Dallas, TX 75390, USA; melissa.huynh@utsouthwestern.edu (M.H.M.); ebenezer.chinedu-eneh@utsouthwestern.edu (E.C.-E.); sasha.alick-lindstrom@utsouthwestern.edu (S.A.-L.); kan.ding@utsouthwestern.edu (K.D.); ryan.hays@utsouthwestern.edu (R.H.); ghazala.perven@utsouthwestern.edu (G.P.)

**Keywords:** responsive neurostimulation, neuromodulation, drug-resistant epilepsy, post-infectious epilepsy, seizures, viral encephalitis, meningitis, herpes simplex virus

## Abstract

Drug-resistant epilepsy (DRE) secondary to viral brain infections (VBI) may have multiple seizure foci, making it not amenable to surgical resection but could respond to responsive neurostimulation (RNS). We aimed to evaluate characteristics of DRE patients with a VBI history who did or did not respond to RNS therapy; 9 patients met criteria. Four out of 9 patients were responders to RNS therapy with ≥50% of seizure-frequency reduction at an average 39-month follow-up. Five patients were non-responders, an average 47-month follow-up. Two responders had a prior destructive surgery. Four non-responders had a prior neurosurgery including 1 focal resection, 2 vagus nerve stimulation, and 1 prior RNS. Patients in the responder group had shorter DRE duration prior to RNS placement than in the non-responder group (average 11.0 years versus 14.4 years). Three responders and four non-responders had a history of focal to bilateral tonic–clonic seizures (FBTC) pre-RNS. Post-RNS, all responders and 2 non-responders stopped experiencing FBTC. Our study demonstrates that RNS therapy could be effective in patients with DRE secondary to VBI, even if the patients failed previous surgical intervention(s). Patients in both responder and non-responder groups had reduction of convulsive seizures. These findings should be considered preliminary observations due to a small sample-size.

## 1. Introduction

Viral brain infections (VBI), including herpes simplex virus (HSV), Epstein-Barr virus (EBV) encephalitis and other viral encephalitides, represent a significant cause of acquired neurological injury [1]. Among their most consequential long-term sequelae is the development of drug-resistant epilepsy (DRE). Patients with VBI face an approximately 6.9-fold greater risk of unprovoked seizures relative to the general population, with reported rates of progression to chronic epilepsy ranging from 4% to 20% across studied cohorts [1,2]. This risk is particularly pronounced following HSV encephalitis, which carries a well-established association with severe DRE [2,3].

The clinical intractability of post-VBI epilepsy is rooted in the nature of the underlying brain injury. Unlike epilepsy arising from a discrete, circumscribed lesion, viral encephalitis inflicts diffuse, multifocal, and frequently bilateral neuronal injury [2]. This diffuse injury pattern means that a single, well-defined epileptogenic zone is rarely identifiable, and even when a dominant focus is suspected, the surrounding network is frequently compromised. As a result, surgical outcomes in postencephalitic epilepsy are notably inferior to those seen in other DRE etiologies, with Engel class I seizure freedom reported in approximately 30% of operated post-VBI patients [4,5]. Furthermore, patients with a history of VBI often demonstrated bilateral or discordant ictal onsets on stereo-electroencephalography (SEEG), rendering many of them poor candidates for resective surgery altogether [6].

Given these limitations, neuromodulation has emerged as a relevant therapeutic strategy for post-VBI epilepsy [7]. A systematic review and meta-analysis from the ILAE Surgical Therapies Commission have confirmed meaningful seizure reductions across neuromodulation modalities, including vagus nerve stimulation (VNS, VNS Therapy^®^, LivaNova, Houston, TX, USA), deep brain stimulation (DBS, Medtronic, Minneapolis, MN, USA), and responsive neurostimulation (RNS, NeuroPace, Inc., Mountain View, CA, USA), in broader DRE populations [8].

The RNS system is FDA-approved for adults with drug-resistant focal epilepsy arising from one or two seizure foci. It consists of a cranially implanted neurostimulator connected to depth and/or subdural strip electrodes placed directly at the seizure focus or foci. The device operates as a closed-loop system, continuously monitoring intracranial electrocorticography (ECoG), detecting patient-specific electrographic patterns, and delivering brief responsive stimulation bursts upon detection. Its capacity for simultaneous lead placement at up to two independent targets is particularly relevant in the post-VBI setting, where bilateral or multifocal onset is the rule, rather than the exception. In a pivotal long-term trial, RNS demonstrated sustained and progressive efficacy, with a median seizure frequency reduction of 75% at nine-year follow-up [9]. Continued efficacy has been corroborated in a large multicenter post-approval study [10].

Despite the alignment of RNS with the multifocal epilepsy characteristic of post-VBI injury, clinical data specifically addressing its use in this population are sparse. Large-scale RNS trials have not systematically stratified outcomes by epilepsy etiology, and post-VBI patients are rarely identified as a discrete subgroup in the existing literature [8,9,10]. The present study addresses this gap by characterizing the clinical profiles, RNS lead targeting strategies, and seizure outcomes in a cohort of patients with DRE secondary to VBI who underwent RNS implantation at our center.

## 2. Materials and Methods

### 2.1. Study Design and Setting

This investigation was designed as a retrospective cohort study. We evaluated patients with DRE who had a history of VBI, underwent stereo-electroencephalography (SEEG) evaluation at our institution from 2014 to 2025, and received RNS implantation. Patients were identified, and data were compiled through a review of a prospectively maintained epilepsy surgery database and electronic medical records. The study protocol was approved by the Institutional Review Board.

### 2.2. Patient Selection

Inclusion criteria for patient selection were the following: (1) a documented history of viral brain infection (e.g., encephalitis or meningitis) substantiated by clinical presentation, polymerase chain reaction results if available, cerebrospinal fluid analysis, and/or neuroimaging findings; (2) a definitive diagnosis of DRE, as defined by International League Against Epilepsy (ILAE) criteria, refractory to adequate trials of at least two appropriately chosen anti-seizure medications (ASMs); and (3) confirmed implantation of the RNS system targeting at least one epileptogenic focus at the University of Texas Southwestern Medical Center. Exclusion criteria included a post-RNS implantation follow-up period shorter than six months, a noninfectious etiology of epilepsy, or incomplete seizure or RNS device data. A total of nine patients satisfied the inclusion criteria.

### 2.3. RNS Implantation and Targeting

All participants completed a standardized, comprehensive pre-surgical evaluation. This typically includes long-term video scalp electroencephalography (EEG) monitoring, high-resolution 3T brain magnetic resonance imaging (MRI), functional magnetic resonance imaging (fMRI), positron emission tomography (PET), magnetoencephalography (MEG), and neuropsychological testing. The patients then proceeded to SEEG using a comprehensive implantation strategy guided by the previous non-invasive pre-surgical testing. The consensus decision to recommend RNS placement and ultimate RNS lead targets was then determined during a multidisciplinary epilepsy surgery conference. This decision was based on the convergence of clinical semiology, surface and/or intracranial EEG data, and structural imaging findings, prioritizing the epileptogenic zones associated with the prior viral injury. The RNS neurostimulator, along with depth and/or subdural strip leads, was surgically implanted following established protocols.

### 2.4. Data Collection

Baseline demographic and clinical variables were systematically gathered and consisted of sex, duration of DRE prior to RNS placement (in years), age at RNS implantation, type of VBI, previous neurosurgery or neuromodulation prior to RNS. Additionally, data on SEEG ictal onset localization, RNS implantation sites, reported seizure frequency per month before RNS and at the last RNS clinic appointment, follow-up duration post-RNS, frequency of focal to bilateral tonic–clonic (FBTC) seizures before and after RNS were also collected. Infection-specific data collected included documented viral etiology and acute illness characteristics.

### 2.5. Outcome Measures

The primary endpoint was the change in seizure burden, quantified by seizure frequency at the final follow-up assessment relative to the preimplantation baseline period in the context of pertinent risk factors and clinical characteristics. Treatment response was dichotomized based on the achievement of ≥50% reduction in monthly seizure frequency as responders and <50% reduction in monthly seizure frequency as non-responders. We also compared seizure severity pre implant and at the last follow-up.

### 2.6. Statistical Analysis

Categorical variables are reported as absolute counts and percentages. Changes in seizure frequency were summarized descriptively using patient comparisons, and the overall responder rate for the cohort was calculated. Our study is purely descriptive and not intended to identify predictors of RNS treatment response.

## 3. Results

### 3.1. Cohort Characteristics

Data from nine patients who met the inclusion criteria were analyzed. In this cohort, there were four responders (44.4%) with an average follow-up duration of 39 (range 10–98) months and five non-responders (55.6%) with an average follow-up duration of 47 (range 8–84) months.

The mean age at the time of acute VBI was 24 years old (range 13–35) for the responder group and 22 years old (range 2–36) for non-responders. All nine patients (responders and non-responders) had seizures at the time of VBI diagnosis. All responders and 3 out of 5 (60%) non-responders continued experiencing intractable seizures after VBI diagnosis. Two non-responders had onset of DRE 4 and 9 years individually after HSV encephalitis. The latency from DRE onset to RNS implantation (duration of DRE) was an average 10.8 years (range 3–16) in responders compared to 14.4 years (range 4–32) in non-responders. Among the responder group, 1 responder (25%) had EBV encephalitis and 3 responders (75%) had presumed viral encephalitis (without identified viral pathogen). Among the non-responder group, 3 non-responders (60%) had confirmed HSV encephalitis, and 2 non-responders (40%) had presumed viral encephalitis (without identified viral pathogen).

### 3.2. MRI Characteristics

Among responders, 1 patient (25%) had multilobar encephalomalacia, 1 patient (25%) had unilateral mesial temporal sclerosis (MTS), 1 patient (25%) had unilateral hippocampal malrotation and 1 patient (25%) had no lesions on pre-surgical brain MRI. Among non-responders, 2 patients (40%) had bilateral hippocampal atrophy, and 3 patients (60%) had multilobar encephalomalacia on pre-surgical brain MRI.

### 3.3. Prior Surgical Treatments

In the responder group, two patients (50%) had undergone prior surgical interventions: one patient received a right anterior temporal lobectomy (ATL) supplemented by laser interstitial thermal therapy (LITT) to the right posterior hippocampus and parahippocampal gyrus. Another underwent left-sided LITT to the hippocampus followed by a left ATL. Among non-responders, four patients (80%) had histories of failed neurosurgical or neuromodulatory procedures, including focal cortical resection (one patient), VNS (two patients), and a previous RNS implantation targeting the left anterior nucleus of the thalamus (ANT) and left temporal neocortex (one patient).

### 3.4. SEEG Ictal Onset Localization

The SEEG evaluation in all nine patients showed two or more ictal onsets, prompting a recommendation for RNS in the multidisciplinary epilepsy conference. Among the responder group, ictal onset was discreet and localized to two (3 out of 4 patients) or three (1 out of 4 patients) regions: (1) right posterior hippocampus and right superior temporal neocortex; (2) left and right temporal operculum and right temporal pole; (3) left mesial temporal and left parietal lobe; and (4) bilateral hippocampi.

Among the non-responder group, the ictal onset was less likely to be localized (1 out of 5 patients) but instead broad or localized to multiple cortical regions (4 out of 5 patients): (1) left hippocampus and right fusiform gyrus; (2) broad onset over right anterior insula and frontal operculum and broad onset over bilateral frontal and anterior insula; (3) right middle temporal lobe, right mesial temporal, left temporal operculum, and left superior temporal lobe; (4) right orbito-frontal, right superior and middle frontal gyri, right temporal lobe; and (5) left mesial temporal lobe, left posterior temporal lobe and left supramarginal gyrus.

The extra-temporal cortex was involved in ictal SEEG onset in 1 out of 4 (25%) responders and 3 out of 5 (60%) non-responders.

Patient characteristics, surgical procedures and RNS therapy outcomes are depicted in Table 1. Figures S1 and S2 demonstrate MRI findings and SEEG tracing in a patient-responder and non-responder, respectively.

### 3.5. RNS Implantation and Seizure Outcomes

Anatomical targets for RNS leads were individualized based on SEEG ictal onset localization. Lead configurations for responders included: right hippocampal and temporal neocortical targeting (depth to frontal operculum, strip to superior temporal gyrus); bilateral temporal operculum and right temporal pole targeting (left depth to temporal operculum, right strip to temporal operculum/pole); left mesial temporal and parietal targeting (dual strips to the temporoparietal junction); and bilateral hippocampal depth electrodes. In the non-responder cohort, leads targeted the bilateral hippocampi, bilateral centromedian (CM) thalamic nuclei, or various combinations of mesial temporal, neocortical, and thalamic regions (e.g., right hippocampus with left superior temporal gyrus, or left temporal neocortex with left CM thalamus).

The mean baseline reported seizure frequency prior to RNS for responders was 10.8 seizures (range 8–15) per month, and for non-responders 11.4 seizures (range 3–30) per month. At the most recent follow-up after RNS implantation, responders reported a mean seizure frequency of 0.3 seizures (0–1) per month, and non-responders reported 10.4 (2–30) seizures per month. One responder reported seizure freedom for 2 years at the most recent follow-up. The average follow-up duration was 39 (range 10–98) months for responders and 47 (range 8–84) months for non-responders.

### 3.6. Focal to Bilateral Tonic–Clonic Seizures

A history of focal to bilateral tonic–clonic seizures (FBTC) was present in 3 responders (75%) and 4 non-responders (80%) at baseline. Following RNS therapy, the prevalence of FBTC decreased, with 0 responders and 2 non-responders (40%) continuing to experience convulsive events.

### 3.7. Anti-Seizure Medications

The mean number of ASMs patient-responders took pre RNS was 2.8, range 2–4, and at last RNS follow-up appointment it was 2.5, range 1–4. Two out of four responders received additional ASMs post RNS placement, one patient was taking 4 ASMs pre RNS and one ASM at last RNS follow-up. In one patient, the number and dose of ASMs were the same pre RNS as they were at last RNS follow-up appointment.

The mean number of ASMs the non-responders took pre RNS and at last RNS follow-up appointment was 3 (range 2–5) and 3.6 (range 2–5), respectively. Three out of five non-responders received additional ASMs post RNS placement. In two patients receiving the same ASMs, the medication doses were increased.

RNS responders’ vs. non-responders’ characteristics are summarized in Table 2.

## 4. Discussion

To our knowledge, this is the largest single-center case series of patients with DRE secondary to VBI who were treated with RNS. Published data specifically addressing RNS therapy outcomes in patients with post-VBI DRE are sparse. Large-scale RNS trials have not systematically stratified outcomes by epilepsy etiology, and post-VBI patients are rarely identified as a discrete subgroup in the existing literature [8,9,10]. Srinivasan et al. reported 5 patients with ultra-refractory post-encephalitis epilepsy; 3 out of 5 (60%) were RNS non-responders with seizure reduction 30%, 40% and 40% and follow-up duration of 18 months, 6 years and 2 years, respectively. All of them required additional neuromodulation device placement with subsequent additional seizure frequency reduction [11]. The other published reports on RNS therapy in patients with DRE related to encephalitis were focused on autoimmune conditions [12,13].

We were interested in evaluating this group of patients within our cohort, as DRE secondary to VBI is more difficult to treat with conventional therapies as viral encephalitis can inflict diffuse neuronal damage, rather than a discrete circumscribed lesion. It was our goal to evaluate patient characteristics and outcomes before and after RNS therapy to contribute to the growing body of literature for this patient population.

Interestingly, although we had defined patients as responders with ≥50% seizure reduction, it was noted that all responders reported either seizure freedom or less than 1 seizure per month and thus had a seizure reduction closer to 97–100%, which was much greater than the defined 50% cutoff. This is opposed to non-responders who, on average, had a seizure frequency reduction of 14% (0–33%).

Our study has a very small sample size, and all presented findings should be regarded solely as descriptive observations. Patients in the responders groups had shorter DRE duration prior to RNS placement than patients in the non-responders group, mean 10.8 years vs. 14.4 years in the non-responders group, but the small sample size does not allow us to make definitive conclusions. Overall, more patients in the non-responders group had identified pathogens of VBI, particularly 60% vs. 25% in the responders group, and HSV as etiology of VBI (60% vs. 0% in the responders group). These percentages correspond to the presence of post-VBI multilobar encephalomalacia on brain MRI, particularly 60% in non-responders vs. 25% in the responders. Furthermore, all non-responders had MRI lesions related to their SEEG seizure onset but in the responders group 1 out of 4 (25%) had non-lesional brain MRI. In the non-responders group, all five patients failed previous neurosurgery versus two out of four (50%) in the responders group. In the non-responders group, 4 out of 5 (80%) had more than three seizure foci or broad ictal onset identified with SEEG vs. 1 out of 4 (25%) in the responders group. Extra-temporal involvement in ictal SEEG onset was noted in 3 out of 5 (60%) vs. 1 out of 4 (25%) in the responders group. The SEEG finding affected selection of the thalamic RNS targets in 2 out of 5 (40%) non-responders vs. 0 responders. Since RNS is limited to only two electrodes as part of the device, two electrodes may not be able to fully treat a patient with multifocal (>two independent seizure foci) or broad, ill-defined ictal onset. We might conclude that non-responders had more severe brain damage due to VBI and a more severe DRE course. Neurophysiological features of the seizures, connectivity studies, RNS electrode type and RNS stimulation parameters could also contribute to the response to neuromodulatory therapy [14,15,16,17,18,19,20,21,22].

Of our nine patients who received RNS therapy, the percentage of disabling seizures decreased in both the responder and non-responder groups. In total, 75% of responders and 80% of non-responders had FBTC seizures prior to RNS therapy and only 40% of non-responders had FBTC after RNS. FBTC can be disabling for patients and can increase the risk for Sudden Unexpected Death in Epilepsy (SUDEP), so this decrease in FBTC in both responders and non-responders was encouraging.

In addition, many patients did not receive RNS therapy in isolation. With medication adjustments, there were patients who received concomitant surgical resection or LITT in addition to RNS therapy (50% of responders and 20% of non-responders). This could suggest RNS therapy was not the sole factor for significant seizure reduction in the responders group, but it is widely acknowledged that RNS is an adjunctive treatment to anti-seizure medications with or without other surgical interventions.

We acknowledge the limitations of this study—most significantly, the retrospective nature, single-center study design, and small sample size. Seizure reduction was collected as a comparison of patient-reported seizures pre and post RNS therapy, which introduces recall bias and overall is inevitably associated with a risk of systematic bias. However, publications on RNS therapy outcomes in patients with DRE related to VBI are limited and our study adds to the body of existing literature.

## 5. Conclusions

Our study demonstrated that RNS therapy could be effective in patients with DRE secondary to VBI even if the patients failed the previous surgical intervention(s). Patients in both responder and non-responder groups had a reduction in convulsive seizures. A more severe DRE course post VBI might contribute to less favorable RNS therapy outcomes. However, our findings should be considered preliminary observations. Although the small sample size limits any definitive conclusions, replication of our findings in larger studies might help to optimize counseling of patients with DRE related to VBI regarding RNS therapy outcomes.

## Figures and Tables

**Table 1:** Patient characteristics, surgical procedures and RNS therapy outcomes.

	Patients	Sex, Age at VBI, Age at RNS, DRE Duration Pre RNS (y)	VBI Pathogen/Brain MRI	NSGY Pre RNS	SEEG Ictal Localization	RNS Implantation Site	Sz Frequency per Mo Pre-RNS/Post-RNS/F/U Duration, Mo	Hx of FBTC Pre RNS/Post RNS (Y/N)	ASMs Pre RNS/at Last RNS F/U, *n*
Responders (≥50% seizure reduction)	1.	F, 25, 34, 9	Unk/R MTS	R ATL, LITT, R posterior hipp and R PHG	R hipp, R T neocortex	Depth to R Fr operculum + strip to R STG	15/0/42	Y/N	4/1
	2.	F, 22, 38, 16	Unk/L hipp malrotation	None	R and L T operculum, R T pole	Depth to L T operculum + strip to R T operculum and R T pole	8/0–1/84	Y/N	2/3
	3.	M, 13, 28, 15	EBV/L FT and R cerebellar encephalomalacia	LITT to L hipp, L ATL	L mesial T and L P	2 strips to L TP junction	12/0/8	N/N	2/2
	4.	M, 35, 38, 3	Unk/Non-lesional	None	L anterior hipp, R anterior hipp	Depths BL hipp	8/0/22	Y/N	3/4
Non-responders (<50% seizure reduction)	5.	F, 35, 39, 4	HSV/BL hipp atrophy, L FP meningioma	VNS	L hipp + R fusiform gyrus	Depths BL hipp	11/8/98	Y/N	5/5
	6.	F, 2, 23, 17	HSV/Multiple small regions of encephalomalacia in R Fr lobe	L T lobe, R insular, R Fr operculum resection	Broad bilateral onset with slight lead in R anterior insula/Fr operculum	Depths BL CM	30/30/10	N/N	3/4
	7.	M, 36, 45, 9	Unk/BL hipp atrophy	VNS	Multifocal BL T mesial and neocortical	Depth R hipp + strip L STG	4/3/50	Y/Y	2/3
	8.	M, 15, 34, 10	HSV/Cystic encephalomalacia RT and insula, R hipp atrophy	None	Multifocal—R OF, R SFG and SMG, RT	Strips R T resection edge + R OF cortex	9/9/51	Y/N	3/4
	9.	F, 24, 56, 32	Unk/ Cystic encephalomalacia of LT, L insula, BL O, BL P	Prior RNS to L ANT + strip electrode over L temp neocortex	LT (hipp, amyg, PHG), L SMG	Strip L T + depth L CM	3/2/28	Y/Y	2/2

Amyg = amygdala, ANT = anterior nucleus of thalamus, ASMs = anti-seizure medications, ATL = anterior temporal lobectomy, BL = bilateral, CM = centromedian nucleus of thalamus, DRE = drug-resistant epilepsy, EBV = Epstein–Barr Virus, F = female, FBTC = focal to bilateral tonic–clonic, Fr = frontal, FP = fronto-parietal, FT = fronto-temporal, F/U = follow-up, HSV = herpes simplex virus, Hipp = hippocampus, Hx = history, L = left, LITT = laser interstitial thermal therapy, M = male, mo = month, MRI = magnetic resonance imaging, MTS = mesial temporal sclerosis, N = no, n = number, NSGY = neurosurgical intervention, OF = orbito-frontal, P = parietal, PHG = parahippocampal gyrus, R = right, RNS = responsive neurostimulation, SEEG = stereo-encephalography, SFG = superior frontal gyrus, SMG = supramarginal gyrus, STG = superior temporal gyrus, Sz = seizure, T = temporal, TP = temporo-parietal, Unk = unknown pathogen, VBI = viral brain infection, VNS = vagal nerve stimulator, Y = yes, y = year.

**Table 2:** RNS responders’ vs. non-responders’ characteristics.

	Responders, *n* = 4	Non-Responders, *n* = 5
Mean age at VBI, y	24	22
Seizure at time of VBI, *n* (%)	4 (100%)	5 (100%)
DRE onset immediately after VBI, *n* (%)	4 (100%)	3 (60%)
Mean latency from DRE onset to RNS placement, y	10.8	14.4
Identified viral pathogen of VBI, *n* (%)	1 (25%)	3 (60%)
HSV as VBI etiology, *n* (%)	0	3 (60%)
Mean pre-RNS seizure frequency per month (range)	10.8 (8–15)	11.4 (3–30)
Patients who had FBTC pre RNS, *n* (%)	3 (75%)	4 (80%)
Patients with documented non-epileptic events, *n* (%)	1 (25%)	1 (20%)
Mean ASM number pre RNS	2.8	3
Brain MRI		
Post-VBI multilobar encephalomalacia, *n* (%)	1 (25%)	3 (60%)
MTS, *n* (%)	1 (25%)	0
Unilateral hippocampal malrotation, *n* (%)	1 (25%)	0
Bilateral hippocampal atrophy, *n* (%)	0	2 (40%)
Non-lesional, *n* (%)	1 (25%)	0
Previous neurosurgery		
Total	2 (50%)	4 (80%)
Resection +/− LITT, *n* (%)	2 (50%)	1 (20%)
VNS, *n* (%)	0	4 (40%)
Previous RNS, *n* (%)	0	1 (20%)
SEEG ictal onset		
2 foci	3 (75%)	1 (20%)
>2 foci or broad	1 (25%)	4 (80%)
Extra-temporal involvement	1 (25%)	3 (60%)
RNS		
Cortical targets, *n* (%)	4 (100%)	3 (60%)
Bilateral thalamic targets, *n* (%)	0	1 (20%)
Cortico-thalamic targets, *n* (%)	0	1 (20%)
Average follow-up duration (range), months	39 (10–98)	47 (8–84)
Mean seizure frequency at last follow-up (range), months	0.3 (0–1)	10.4 (2–30)
FBTC at last RNS follow-up, *n* (%)	1 (25%)	2 (40%)
Mean ASM number at last follow-up	2.5	3.6

ASMs = anti-seizure medications, DRE = drug-resistant epilepsy, FBTC = focal to bilateral tonic–clonic seizures, HSV = herpes simplex virus, LITT = laser interstitial thermal therapy, MTS = mesial temporal sclerosis, *n* = number, RNS = responsive neurostimulation, SEEG = stereo-electroencephalography, VBI = viral brain infection, VNS = vagus nerve stimulation, y = years.

## Data Availability

The original contributions presented in this study are included in the article. Further inquiries can be directed to the corresponding author.

## Supplementary Materials

The following supporting information can be downloaded at https://www.mdpi.com/article/10.3390/neurosci7030068/s1: Figure S1: Patient 3 with DRE post-EBV encephalitis, responder to RNS therapy; Figure S2: Patient 9 with DRE post-HSV encephalitis, non-responder to RNS therapy.
