# Supplementary material for: Responsive Neurostimulation in Patients with a History of Viral Brain Infections—A Single-Center Experience
Source: NeuroSci. 2026 Jun 13;7(3):68. doi: 10.3390/neurosci7030068 (PMC13304574; doi:10.3390/neurosci7030068)
Supplement: Supplementary file 1 [file neurosci-07-00068-s001.zip › neurosci-4375626-supplementary.pdf]

Brief Report

Responsive Neurostimulation in Patients with a History of Viral Brain Infections—A Single-Center Experience

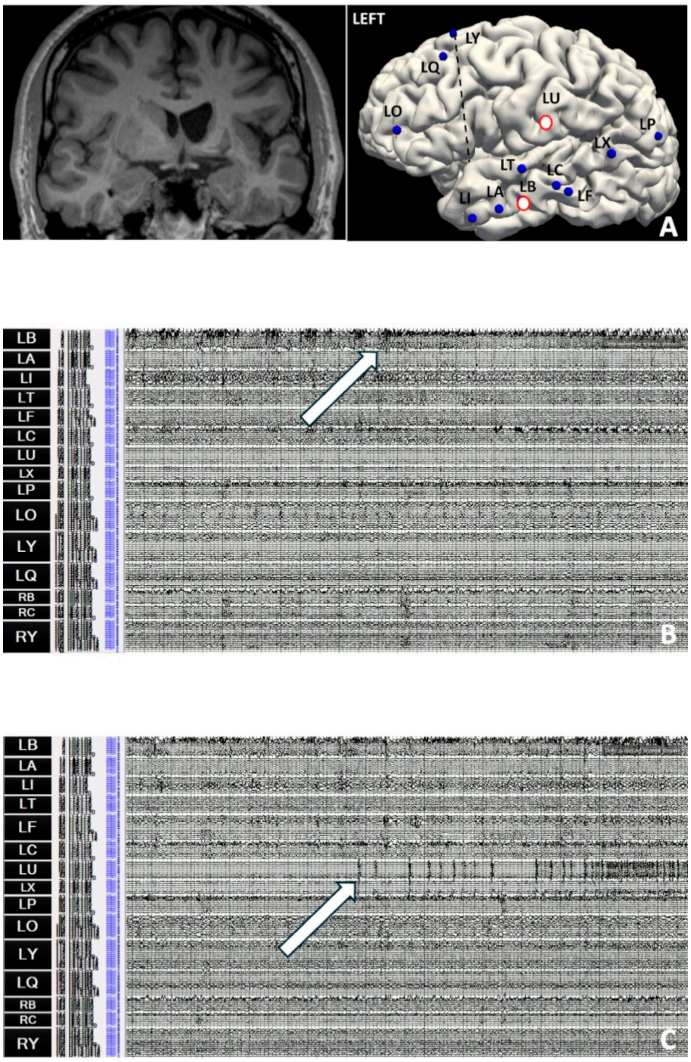

Figure S1. Patient 3 with DRE post-EBV encephalitis, responder to RNS therapy. (a) Left, brain MRI showed asymmetric left frontotemporal atrophy. Right, SEEG ictal onset map. The circles indicate the ictal onset 1 localized to the left hippocampus sampled with LB1-2 electrode contacts, and the ictal onset 2 localized to the left parietal operculum sampled with LU1-10 electrode contacts. (b) SEEG tracing

depicting the ictal onset 1 localized to the left hippocampus sampled with LB1-2 electrode contacts (arrow). Sensitivity 75 microvolts, low-frequency filter 5.3 Hz, high-frequency filter 300 Hz, 30-second page, bipolar montage. (c) SEEG tracing depicting the ictal onset 2 localized to the left parietal operculum sampled with LU1-10 electrode contacts (arrow). Sensitivity 75 microvolts, low-frequency filter 5.3 Hz, high-frequency filter 300 Hz, 30-second page, bipolar montage.

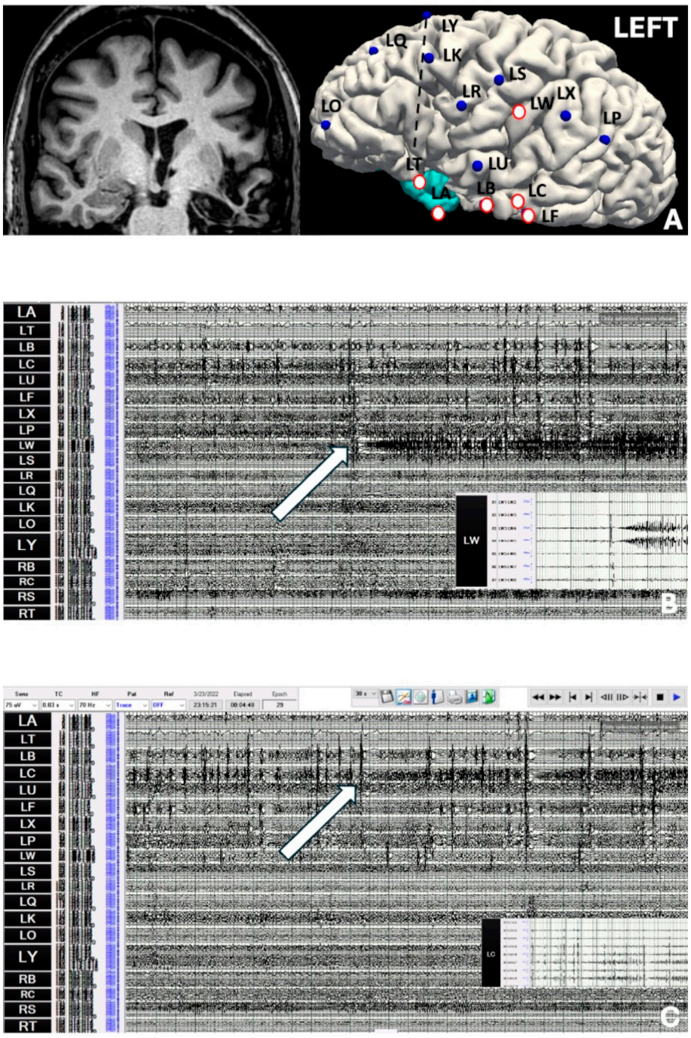

Figure S2. Patient 9 with DRE post-HSV encephalitis, non-responder to RNS therapy.

(a) Left, brain MRI showed multifocal encephalomalacia with marked atrophy of the left temporal lobe and hippocampus. Right, SEEG ictal onset map. The circles indicate the ictal onset 1 localized to the left supramarginal gyrus sampled with LW4-5 electrode contact, the ictal onset 2 localized to the left middle

temporal gyrus sampled with LC6-8 electrode contacts, the ictal onset 3 localized to the left amygdala remnant and middle temporal gyrus near encephalomalacia sampled with LB4-5 and LA1-3 electrode contacts, the ictal onset 4 localized to the left inferior temporal gyrus sampled with LF6-8 electrode contacts, the ictal onset 5 localized to the left fusiform gyrus sampled with LF1-3 electrode contacts, and the ictal onset 6 localized to the left amygdala remnant and left superior temporal gyrus near encephalomalacia sampled with LA1-3 and LT1 electrode contacts. (b) SEEG tracing depicting the ictal onset 1 localized to the left supramarginal gyrus sampled with LW4-5 electrode contact (arrow and zoomed, bottom right). Sensitivity 75 microvolts, low-frequency filter 5.3 Hz, high-frequency filter 300 Hz, 30-second page, bipolar montage. (c) SEEG tracing depicting the ictal onset 2 localized to the left middle temporal gyrus sampled with LC6-8 electrode contacts (arrow and zoomed, bottom right). Sensitivity 75 microvolts, low-frequency filter 5.3 Hz, high-frequency filter 300 Hz, 30-second page, bipolar montage.
